# Generation of Monoclonal Antibody MS17-57 Targeting Secreted Alkaline Phosphatase Ectopically Expressed on the Surface of Gastrointestinal Cancer Cells 

**DOI:** 10.1371/journal.pone.0077398

**Published:** 2013-10-15

**Authors:** Ming Li, Jianpeng Gao, Runhua Feng, Yuling Wang, Xuehua Chen, Jianyu Sun, Dongqing Zhang, Zhenggang Zhu, Lee M. Ellis, Mason Lu, Jeffrey E. Lee, Zhenqing Feng, Bingya Liu

**Affiliations:** 1 Department of Pathology, Nanjing Medical University, Nanjing, Jiangsu Province, China; 2 Shanghai Key Laboratory of Gastric Neoplasms, Shanghai Institute of Digestive Surgery, Ruijing Hospital, Shanghai Jiaotong University School of Medicine, Shanghai, China; 3 Department of Surgical Oncology, University of Texas MD Anderson Cancer Center, Houston, Texas, United States of America; 4 Shanghai MabStar, Inc., Shanghai, China; 5 Shanghai Institute of Immunology, Shanghai Jiaotong University School of Medicine, Shanghai, China; The University of Hong Kong, Hong Kong

## Abstract

**Background:**

Therapeutic antibody development is one of the fastest growing areas of the pharmaceutical industry. Generating high-quality monoclonal antibodies against a given therapeutic target is crucial for successful drug development. However, due to immune tolerance, making it difficult to generate antibodies using conventional approaches.

**Methodology/Findings:**

Mixed four human gastric cancer (GC) cell lines were used as the immunogen in A/J mice; sixteen highly positive hybridoma colonies were selected via fluorescence-activated cell sorting-high throughput screening (FACS-HTS) using a total of 20,000 colonies in sixty-seven 96-well plates against live cells (mixed human GC cells versus human PBMC controls). MS17-57 and control commercial Alkaline Phosphatase (ALP) mAbs were used to confirm the target antigens (Ags), which were identified as ALPs expressed on the GC cell surface through a combination of western blot, immunoprecipitation and mass spectrometry (MS).

MS identified the Ags recognized by MS17-57 to be two variants of a secreted ALP, PALP and IALP (Placental and intestinal ALP). These proteins belong to a hydrolase enzyme family responsible for removing phosphate groups from many types of molecules. Immunofluorescence staining using MS17-57 demonstrated higher staining of gastrointestinal (GI) cancer tissues compared to normal GI tissues (*P*<0.03), and confirmed binding of MS17-57 to be restricted to a functional epitope expressed on the cancer cell surface. Proliferation assays using the PALP/IALP-expressing GC cell lines demonstrated that MS17-57 inhibited cell growth by 32±8%. Transwell cell migration assays documented that MS17-57 can inhibit PALP/IALP-expressing GI cancer cell migration by 25±5%. MS17-57 mAb inhibited tumor growth in nude mice.

**Conclusions:**

Our findings indicate that PALP and IALP can be ectopically expressed on extracellular matrix of GI cancers, and that MS17-57 directed against PALP/IALP can inhibit GI cancer cells growth and migration *in* *vitro* and *in* *vivo*. This investigation provides an example of identification of cancer biomarkers representing promising therapeutic targets using mAb generated through a novel HTS technology.

## Introduction

Gastrointestinal (GI) cancer is one of the most common malignant tumors in humans with a high risk of mortality worldwide [1]. The development of therapeutic antibodies to investigate, evaluate, prevent and treat cancer is one of the fastest growing areas of research in both the academic arena and the pharmaceutical industry [2]. The generation of high-quality monoclonal antibodies (mAbs) against cancer markers as therapeutic targets is an important avenue for clinical drug development. Some cancer markers are known (e.g., human epidermal growth factor receptor 2 and vascular endothelial growth factor) or are well developed, but most are still unknown or undeveloped [3]. There is therefore great interest in generating mAb against novel and unknown cancer targets. mAb against specific tumor targets can be developed and identified using a variety of approaches, including enzyme-linked immunosorbent assay (ELISA)-high throughput screening (HTS), fluorescence-activated cell sorting (FACS)-HTS, and *in vitro* and *in vivo* screening.

Identification of novel cancer biomarkers involved in tumorigenesis, cancer development, or cancer prevention continues to be of great interest worldwide [4,5]. Due to advances in proteomics and other aspects of molecular biology, such investigations are increasingly more feasible in current era than in the past. Cutting-edge HTS technology is relatively well developed and is very popular in many academic fields [6,7].

We therefore have investigated the generation of mAbs against potentially novel Ags on the cancer cell surface using a FACS-HTS method. In this study, we found that MS17-57 mAbs could identify placental and intestinal alkaline phosphatases (PALP and IALP, respectively) as targets expressed on the cancer cell membrane. Our strategy was to exploit a novel method of FACS-HTS and hybridoma technology using a mixture of 4 live GI cancer cell lines as immunogen [8], hypothesizing that at least some of the mAb produced would be likely to bind to conformational epitope(s) on the cell surface of GI cancer cells. The data demonstrated that MS17-57 could bind to PALP and IALP that were ectopically expressed on cell surface, and could neutralize ALP activity both *in vitro* and *in vivo*. These results suggest that PALP and IALP expressed on the GI tumor surface with aberrant cancer cell metabolism and signaling pathway in which they may promote cancer cell growth and metastasis. MS17-57 is a mAb with high affinity and specificity, potentially representing a useful reagent and a potential basis for a novel therapeutic strategy in cancer drug development.

Our future studies will focus on the molecular mechanism of MS17-57 inhibition of cancer cells proliferation and migration via binding to PALP/IALP on the cancer cell surface, and on clarifying intracellular signaling pathways affected by the action of this antibody. Presuming additional investigations confirm the promising results described in these preliminary investigations, antibody engineering of MS17-57 as a chimeric or humanized antibody for therapeutic application could be pursued. 

## Materials and Methods

### Cell Cultures

GI cancer cell lines MKN45, BGC823, SGC7901, MKN28, AGS and GES-1 were purchased from the Institute of Biochemistry and Cell Biology, Shanghai Institutes for Life Science, Chinese Academy of Science (Shanghai, China) and the Riken BioResource Center (Tsukuba, Japan). The GI cancer cell lines MKN-74 [9] and TMK-1 [9] were the courtesy of Dr. Gary K. Schwartz (Memorial Sloan-Kettering Cancer Center, New York, NY, USA), and KKLS cells [10] were obtained from Dr. Yukuta Takahashi (Cancer Research Institute, Kanazawa University, Kanazawa, Japan). The gastric cancer cell lines ST-8 and ST-9 [11] were the courtesy of Drs. Bradley McIntyre and Paul F. Mansfield, the University of Texas MD Anderson Cancer Center (Houston, TX, USA). All other cell lines (SP2/0, etc.) were obtained from the American Type Culture Collection (Manassas, VA, USA). Peripheral blood mononuclear cells (PBMCs) were obtained from a healthy volunteer with informed consent.

Except for SP2/0 myeloma cells and mAb hybridoma cells, all cells were cultured in MD6, a homemade, serum-free medium derived from Dubecco’s modified Eagle’s medium (Gibco BRL, Rockville, MD, USA) with 5% heat-inactivated fetal bovine serum (FBS) (Sigma, St. Louis, MO, USA), 100 units/mL penicillin, and 100 µg/mL streptomycin (Invitrogen, Carlsbad, CA, USA). The myeloma and hybridoma cells were cultured similarly but without fetal bovine serum. Myeloma and hybridoma cells were grown in suspension. GI cancer cells were grown to adhere to tissue culture flasks with 5% FBS/MD6 medium.

### Patient Tissues

 Fresh tumor specimens and adjacent noncancerous tissues (mucosa) of seven GC patients undergoing surgery were obtained from the Department of Surgery of Shanghai Ruijing Hospital at Shanghai, China. These patients had not received cytotoxic chemotherapy or radiotherapy prior to surgery. Institutional Review Board protocols were observed, and ethical approval for the study was granted by the Research Ethics Committee of Ruijing Hospital, Shanghai Jiaotong University School of Medicine. Informed written consent had been obtained from all participants (including voluntary donors) involved in the study.

 Fresh tissues were stained with MS17-57 as well as isotype control mAb. The binding signals were amplified, labeled with fluorescein isothiocyanate, and counted by FACS-HTS.

### Mice

 Male A/J mice (The Jackson Laboratory, Bar Harbor, ME, USA) 6–8 weeks old were purchased from the Nanjing Experimental Model Animal Center, Nanjing University, Nanjing, China, and maintained at the animal facility of Shanghai MabStar, Shanghai, China. The maintenance of A/J mice and experimental procedures were approved by Shanghai Animal Welfare and Research Ethics Committee, Science and Technology Committee of Shanghai Municipal Government, China and the license number is SYXK(Hu)2009-0087.

Male JAX nude mice aged 4–8 weeks were purchased from the Shanghai Experimental Model Animal Center and maintained at the Experimental Animal Center of Shanghai Ruijing Hospital. These nude mice were used for experiments with xenografts of human gastric cancer (GC) and the methods are similar to Sela group previously described [12]. The maintenance of JAX nude mice and experimental procedures were approved by Shanghai Animal Welfare and Research Ethics Committee, Science and Technology Committee of Shanghai Municipal Government, China and the license number is SYXK(Hu)2011-0113.

### Live Cancer Cell Immunization

Each A/J mice was injected subcutaneously at 5–10 sites as well as once intraperitoneally (i.p.) with about 5–10x10^6^ cells (equal amounts of MKN45, BGC823, SGC7901, and MKN28 cells) in phosphate-buffered saline (PBS; pH7.4). Three mice were assigned per experimental batch. Two weeks later, the mice were injected again, for a total of three immunizations, plus one i.p. booster three days before the fusion experiment.

Three days after the booster, spleen cells were collected by surgery from an immunized mouse sacrificed by CO_2_ inhalation and all efforts were taken to reduce animal suffering; the spleen cells were then fused with myeloma SP2/0 cells [13,14] in 50% polyethylene glycol (pH7.4) fusion solution to generate mAb hybridomas. The fused hybridoma cells were maintained in hypoxanthine-aminopterin-thymidine medium (Sigma-Aldrich, St. Louis, MO, USA), and 280 µL/well fused cells (about 1–2 x 10^4^ spleen cells/well) were aliquoted into sixty seven 96-well flat-bottomed tissue culture plates and incubated at 37°C in an incubator with 5% CO_2_ for 10 days. At the same time, the four GI cancer cell lines were grown in bulk in multiple flasks. 

### Fluorescence-Activated Cell Sorting with High-Throughput Screening (FACS-HTS)

The FACSCalibur-HTS system (Becton Dickinson, San Jose, CA, USA) was used to screen the selective hybridomas from large amounts of fusion cells/colonies in the fusion plates. Up to 200 screening and counter screening (cancer cells versus normal cells) plates could be used in such a screening assay. Cells from all four GC lines were harvested, mixed, and aliquoted (about 1x10^6^ cells/well)into 96-well U-bottom plates (67 plates).The same number of human peripheral blood mononuclear cells (PBMCs) from a healthy volunteer were separated by using the human lymphocyte-separating solution Ficoll-Plaque Plus(GE Healthcare Biosciences, Pittsburgh, PA, USA) and aliquoted into 96-well U-bottom plates (another 67 plates for counter screening). The supernatant (80 µL/well)from each of the 67 fusion plates was transferred to GC cell plates labeled from 1 to 67 after these cells had been blocked by 2.0% bovine serum albumin (BSA)/PBS blocking buffer in the plates. Supernatant from the same fusion plates were similarly transferred to PBMC plates. After the GC cell and PBMC plates were washed three times with blocking buffer, the secondary antibody [goat anti-mouse immunoglobulin G (IgG) Fc conjugated with fluorescein isothiocyanate] was aliquoted into both GC cell plates and PBMC plates (100 µL/well) and incubated on ice or in a refrigerator at 4°C for 30 minutes, and washed again three times with blocking buffer. The fluorescent stained cells were fixed in 1.5% paraformaldehyde/PBS. The percentage of stained cell peak shift and mean fluorescence intensity (MFI) were continuously monitored by the FACS-HTS system for 134 screening and counter-screening 96-well U-bottom plates. Hybridoma colonies exhibiting strong binding and specificity for GC cells (and no binding to PBMCs) were selected for expansive growth, weaned from conditioned medium, and subcloned.

### mAb Generation

Supernatants were collected from the selected hybridoma clones and purified using Protein-A Sepharose columns (Sigma-Aldrich, St. Louis, MO, USA). We chose the purified MS17-57 mAb for additional analysis, which was filtered through a 0.2-µm membrane, sterilized, and aliquoted into cryotubes kept at 4°C for use in cell cultures or *in vivo* studies (described below). The mixture of mAb in PBS and 50% glycerol was frozen at −20°C for long-term storage.

### Mouse IgG Isotyping

We used a mouse mAb isotyping kit (IsoStrip, RochePharma AG, Reinach, Switzerland) to characterize the isotype of the mouse MS17-57 mAb (IgG).

### cDNA Sequencing of the Variable Region of MS17-57

We used an RNeasy kit (Qiagen, Valencia, CA, USA) to isolate total RNA from MS17-57 hybridoma cells. The MS17-57 cDNA library was created from mRNA in reverse transcription reactions with a SuperScript III first-strand kit (Invitrogen, Grand Island, NY, USA). The MS17-57 IgG Fab fragment Ag-binding variable regions were amplified by polymerase chain reaction (PCR) with 21 pairs of heavy-chain and light-chain primers, which were obtained from the Mouse IgG Library Primer Set (Progen Biotechnik, Heidelberg, Germany). PCR products were used for DNA sequencing, which was performed by the Lee & Lu lab at the MD Anderson Cancer Center, Houston, TX, USA. Complementarity-determining regions (CDRs) and framework regions (FWRs) of MS17-57 were identified using resources available at the National Center for Biotechnology Information websites and determining the alignments of cDNA and amino acid sequences [15-18].

### Indirect ELISA

Ag (protein) (0.2 µg/mL in PBS) was coated onto Immulon-II HB 96-well ELISA plates (Thermo Fisher Scientific, Waltham, MA, USA) and incubated in a wet-box overnight at room temperature (RT). Ag-coated plates were washed and blocked by 1.0% BSA/PBS-Tween 20 (PBST) buffer, and 100 µL of primary antibodies individually diluted in 1.0% BSA/PBST were added to each well. The plates were incubated for 1 hour at RT and washed with PBST. After washing, 100 µL of diluted (1:2,500) horseradish peroxidase (HRP)-conjugated goat anti-mouse IgG Fc polyclonal secondary antibody (Jackson ImmunoResearch Laboratories, West Grove, PA, USA) was added to each well and incubated for 1 hour at RT. After an additional wash with PBST, 150 µL of peroxidase substrate (tetramethylbenzidine in 0.02M [pH6.0]citrate/acetate buffer and 0.003% H_2_O_2_) was added to each well to develop the color of binding signals; development was stopped by adding 50 µL of 0.2M H_2_SO_4_ to each well. The absorbance (optical density; OD) of the reaction plates was read at 450 nm with the turbidity reference set at 620nm. 

### Immuocytochemical Analysis with Cytospin Slides

 To make 1x10^6^ GC cells in 50 µL/each, cytospin chamber holes were spun onto slides and fixed with 4% paraformaldehyde/PBS solution, dehydrated with 70% ethanol and air dried. Slides were rehydrated in PBST in a flat position for 5 minutes and then incubated in 10% goat serum/PBS. Slides were incubated with primary antibodies at an appropriate dilution for 1 hour at RT or overnight at 4°C, rinsed in PBST twice for 5 minutes/each in a horizontal position. Slides were then incubated with the HRP-labeled secondary antibody (goat anti-mouse IgG Fc–HRP, Jackson ImmunoResearch Laboratories) at 1:500 dilution in PBS for 30 minutes at RT. Detection the mAb staining on cancer cells was performed with 0.125% aminoethylcarbazole chromogenic substrate for 5–10 minutes at RT, and the mAb stained cytospin slides were counterstained with Gill’s hematoxylin (Dako, Carpinteria, CA, USA). Anti-fade mounting medium (Vector Labs, Burlingame, CA, USA) was used to mount the slides.

### Cancer Cell Proliferation Inhibition Assay

 The cancer cell proliferation inhibition assay was conducted using Cell Counting Kit-8 (Dojindo Molecular Technologies, Santa Clara, CA, USA) and was based on the detection of dehydrogenase activity in viable cells. A total of 5,000 cells/150 μL of selected BGC823, MKN45, or both types of cells from 4 GC cell lines used in immunization, was dispensed in each well of 96-well plates for each of quadruplicated test conditions. Plates were pre-incubated for 24 hours in a humidified incubator at 37°C with 5% CO_2_. 50 μL/well of MS17-57 (8 and 2 µg/mL), an irrelevant (isotype control) mAbs (30 and 8 µg/mL) and one medium alone (blank control) was added to the test plates in quadruplicate to reduce variation. The test plates were incubated using the same conditions as the pre-incubation. CCK-8 solution was added to the plates (10 μL/well), taken as one plate per each test condition per day from days 1 to 7. The plates were incubated for 3 hours and the absorbance at 450 nm was measured using a VERSAmax microplate reader (Molecular Devices, Sunnyvale, CA, USA).

### Cancer Cell Migration Inhibition Assay

The inhibition of chemotaxic cancer cell migration was assessed using the QCM 24-well colorimetric cell migration assay (EMD Millipore, Billerica, MA, USA). At RT, 300 μL of the cell suspension (1.0 x 10^5^ cells/mL in MD6), with or without each 5, 10, and 20 µg/mL MS17-57 plus irrelevant mAbs added to each insert, was added to each of the 24 wells of the migration chamber, and 500 μL of MD6 with 5% fetal bovine serum was added to each well of the lower chamber. The test plates were incubated in a tissue culture incubator at 37°C with 5% CO_2_ for 24 hours, and non-migrating cells were gently removed from the interior of the inserts with a cotton-tipped swab. Cells on the lower surface of the membrane were stained by dipping the inserts in crystal violet (a nucleic dye) staining solution (500 μL/well)for 20 minutes. The inserts were rinsed in water several times, allowed to air dry, and photographed. The dyed cells were aliquoted into 96-well plate and counted by VERSAmax microplate reader (Molecular Devices, Sunnyvale, CA, USA).

### Tumor Growth in Balb/C Nude Mice

 The antitumor activity of the MS17-57 was studied with xenografts of human GC in Balb/C nude mice as previously described [12]. Briefly, 1 x 10^6^ selected BGC823 or MKN45 cells in MD6 with or without MS17-57 plus irrelevant mAbs were injected i.p. into each mouse. This injection produced tumors in all the mice by 6 weeks after cell implantation, and the weight of the tumor nodule of each was about 0.3–1.0 gram, which depends on how many initial cells were inoculated. Treatment with MS17-57, irrelevant mAbs, and PBS (as medium blank controls) (all i.p.) started the day after cancer cell injection (i.e., at day 1) and was repeated at days 15 and 29. Each treatment group consisted of at least four animals. The number of tumor nodules was counted, and the nodule diameter was measured once. The mice were sacrificed by CO2 inhalation at day 48 and all efforts were made to ameliorate animal suffering. 

### Immunoprecipitation (IP) and Mass Spectrometry (MS) Analysis

For indirect IP, magnetic Dynabeads coated with protein-A (Invitrogen, Grand Island, NY, USA) were incubated with 50 µg of MS17-57 for 30 minutes at RT with constant shaking. The beads were then washed and incubated with lysate of selected BGC823 or MKN45 GC cells, which the lysates were diluted appropriately. Incubation occurred in the presence of 0.1% TritonX-100 in radioimmunoprecipitation assay extracts (Sigma-Aldrich, St. Louis, MO, USA). mAb against anti-β-actin (about 42 kDa in sodium dodecyl sulfate-polyacrylamide gel electrophoresis [SDS-PAGE] gel) was used as an internal calibration standard. The lysate was successively exposed to MS17-57–coated beads for 30 minutes with rotation. Bound beads were washed with 0.5% TritonX-100 followed by water. The samples were eluted by heating in boiled water with 50 µL/each eluate of Laemmli denaturing sample buffer (Bio-Rad, Hercules, CA, USA). Then the samples were loaded and separated on 10% Bis-Tris gel (Bio-Rad) in 1× 2-(*N*-morpholino)ethanesulfonic acid running buffer and SDS-PAGE. The gel was stained with a silver stain kit (Invitrogen, Grand Island, NY, USA). The stained target bands were cut and analyzed with a ProteinChip system series 4000 (Enterprise Edition) mass spectrometer (Bio-Rad).

Direct IP was performed using the Dynabeads antibody coupling kit (Invitrogen, Grand Island, NY, USA) to directly couple the beads with MS17-57. The remaining steps were the same as described for indirect IP.

### mRNA Expression by Quantitative Reverse Transcription (qRT)-PCR Analysis

 Extraction of total RNA from 10 GI cancer cell lines was performed with TRIzol reagent (Invitrogen, Grand Island, NY, USA) [19]. RNA was quantified using the A260/A280-nm absorption ratio. To convert RNA into cDNA, a 1 µL of the total RNA sample was used in a reverse transcription reaction with RNase inhibitor (Invitrogen), SuperScript III reverse transcriptase (Invitrogen), dithiothreitol, and first-strand buffer. The mixtures of the samples were incubated at RT for 10 minutes and then at 42°C for 50 minutes. The reaction was deactivated at 70°C for 15 minutes.

 The forward and backward primers of IALP, PALP, and glyceraldehyde-3-phosphate dehydrogenase(5'-TCCATCTTCGGGTTGGCCCCC-3' and 5'-TCCGTGGGTCTCGGACGACAG-3'; 5'- CCTGGGTGCTGCTCCTGCTGGG-3' and 5'-CGTAGACACCCCCATCCCGTCAC-3'; and 5'-GGACCTGACCTGCCGTCTAG-3' and 5'-GTAGCCCAGGATGCCCTTGA-3', respectively)were synthesized and used in a real-time PCR reaction with SYBR green reagents (Life Science, Hercules, CA, USA) and loaded onto a 96-well plate. The mixture of samples was run in a CFX96 Touch real-time PCR detection system (Bio-Rad) under the following conditions: 93°C for 2 minutes for pre-denaturation, 93°C for 1 min for denaturation, 55°C for 1 min for annealing, 72°C for 1 min for extension in 40 cycles, and 72°C for 7 min for the final incubation. Data were analyzed using Opticon Monitor software (Life Science, Hercules, CA, USA).

### Statistical Analysis

Statistical analysis was conducted using SPSS software (IBM). Data were expressed as means ± standard error of the means. Differences were analyzed by Student’s t-test; *P* values <0.05 were considered as significant.

## Results

### MS17-57 Hybridoma Was Generated by Immunization with Live GC Cells

**Figure 1:**
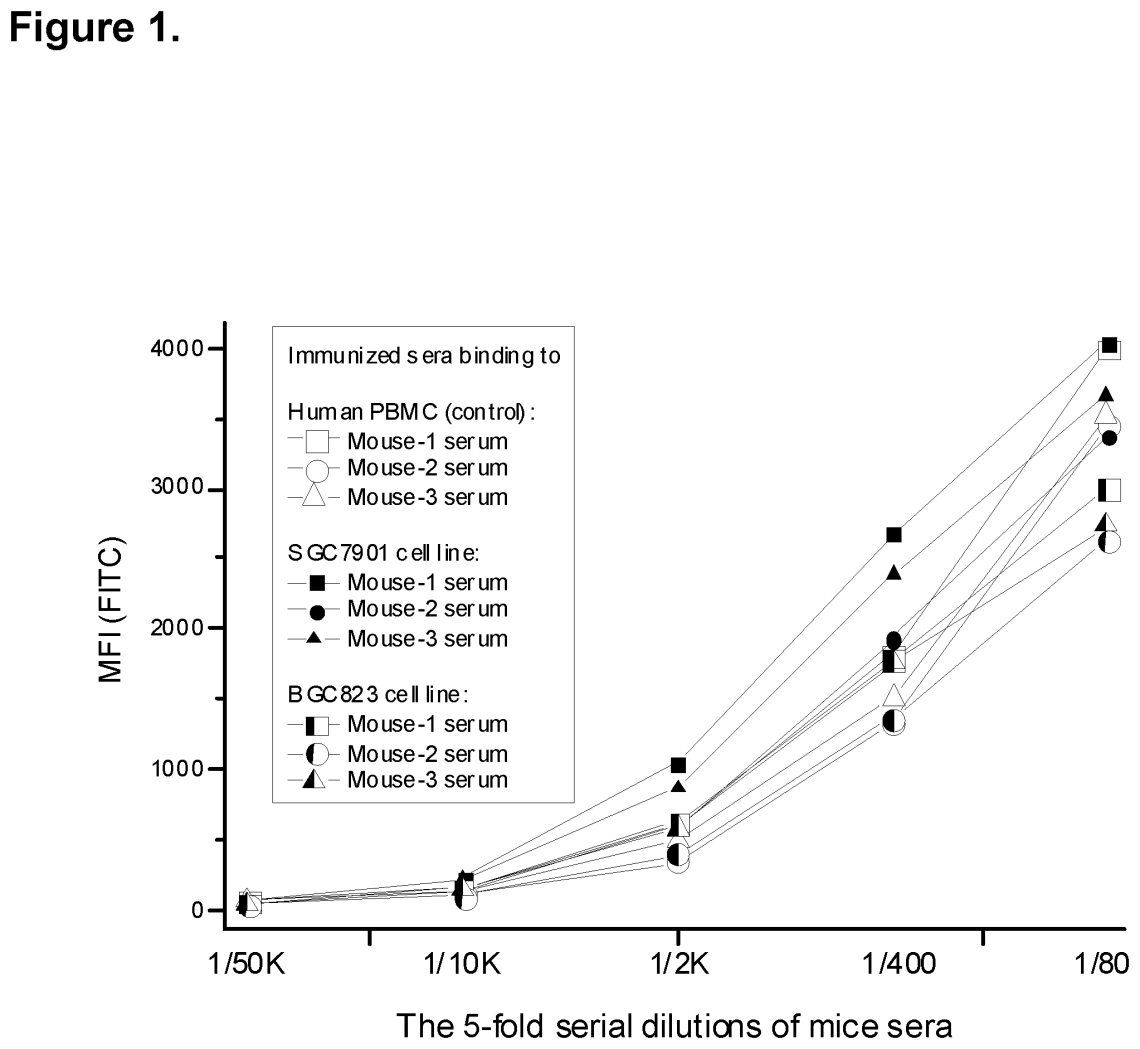
Titration analysis of live GC cells used to immunize mice and bound to mouse serum. Human PBMCs were isolated from whole blood of a selected healthy donor for normal cell control. MKN45, BGC823, SGC7901, and MKN28 cells were used in experiments, but SGC7901 and BGC823 cell lines were randomly selected as examples in this figure. The MFI were higher in these cells than in PBMCs. All three mice exhibited strong immune responses to the human GC cell lines.

Following the principles of mAb generation [10], we used a mix of live MKN45, BGC823, SGC7901, and MKN28 GC cells as the immunogen to immunize A/J mice three times. Before the final boost, the serum from a tail-bleed of each immunized mouse was collected. SGC7901 and BGC823 cells were randomly selected from the four GC cell lines that were used in live cell immunization. Human PBMCs isolated from whole blood of a healthy donor were used as a noncancer control. Human GC cells and PBMCs were bound with serum from each immunized mice and with nonimmunized mouse serum for control (Figure **1**). All three mice in each cell line group exhibited binding signals to GC cell lines and PBMCs, although PBMCs had relatively weaker signals.

Spleen cells from four human GC cell lines immunized mice were fused with mouse myeloma SP2/0 cells to generate the antibody hybridoma. FACS-HTS was used to screen for stronger Ag binding from a large pool of binders (i.e., mAbs) in the hybridoma repertoire. The mAbs bound mainly to the conformational epitopes on the surface of live cancer cells. Using FACS-HTS, we selected hybridoma colonies with strong binding signals using a total of 20,000 colonies in sixty-seven 96-well plates against the mixed live GC cells and normal human PBMCs (counter screening cells). After these hybridoma cells were weaned from the selection medium, the five hybridomas with the highest binding signals were selected for subcloning and antibody affinity purification. We chose the MS17-57 clone for further study. (The four other mAbs will be assessed at a later date.) 

### MS17-57 Characterization

**Figure 2:**
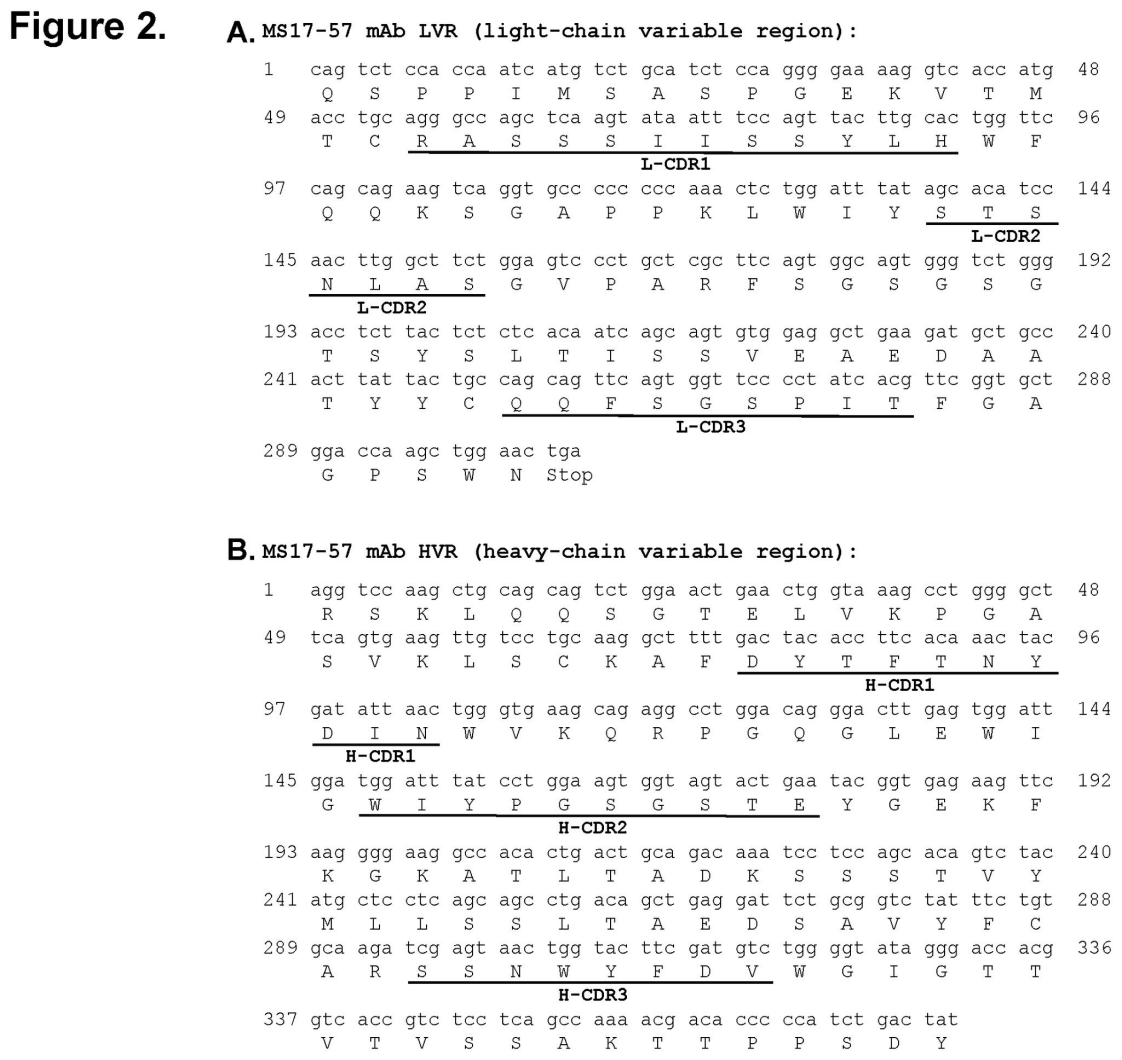
cDNA and amino acid sequences of the variable regions of the MS17-57 light chain (A) and heavy chain (B). FWRs are located between CDRs.

We characterized MS17-57 mAb with a variety of methods. Information on IgG1 for the heavy chain and κ for the light chain was obtained from mouse antibody isotyping [20]. The variable regions (light chain and heavy chain) in the Fab fragment of MS17-57 were sequenced for DNA and amino acids (Figure **2**). The unique Ag-binding regions demonstrated that the CDRs between the FWRs of the heavy and light chains were present in the variable region of Fab fragment as the MS17-57 identity.

### MS17-57 Binding to the Lysates of GI Cancer Cells

**Figure 3:**
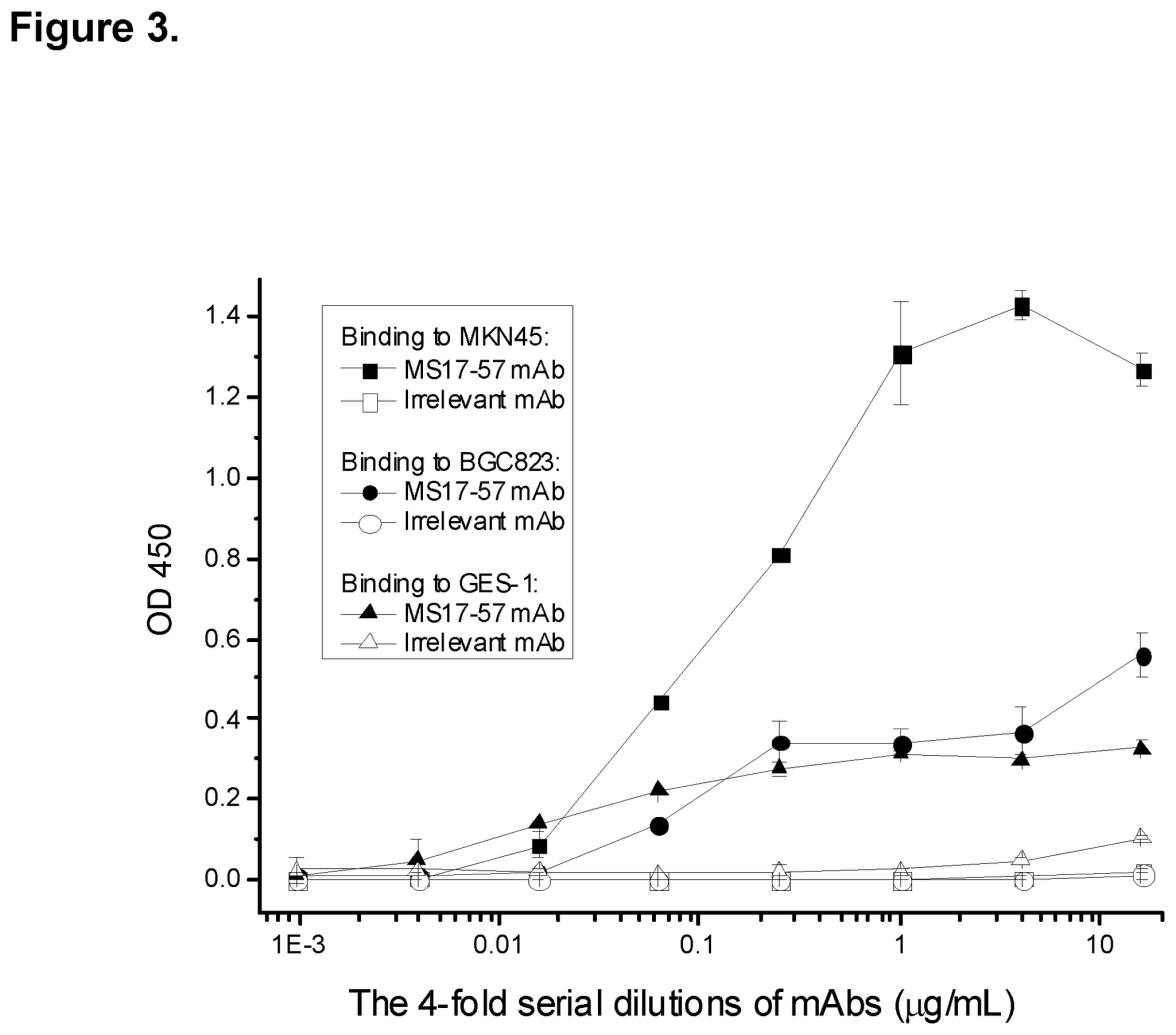
Binding of MS17-57 to lysates of MKN45, BGC823 and GES-1 cell lines in indirect ELISA. MKN45, BGC823, SGC7901, and MKN28 cells were used in experiments, but MKN45 and BGC823 cell lines were randomly selected as examples in this figure. MS17-57 expressed strong binding signals to MKN45 cells and moderate binding signals to BGC823 cells and GES-1 cells. Cell lysates were coated with 1.0 µg/mL PBS onto Immulon-II HB 96-well ELISA plates (100 µL/well). The protein concentrations of these cell lysates were balanced, but not for the binding targets (Ags) that could be a big variation. Irrelevant mAb was used as an isotype control.

To define some biological features of MS17-57 mAb, lysates of MKN45, BGC823, and GES-1 cell lines (the latter generated and immortalized from normal stomach mucosal cells and transformed with Simian virus 40) [21] were individually coated onto ELISA plates. The purified MS17-57 added on the plate plus secondary antibody-HRP amplified the binding signals (Figure **3**). This indirect ELISA showed that MS17-57 could still bind specifically to the denatured target(s) of cell membrane protein from the cancer cell lysates. MKN45 cells expressed the MS17-57 target at a higher level than BGC823 or GES-1 cells did, indicating that target expression levels might differ between cell lines. 

### Localization of MS17-57 Targets on Cancer Cells

**Figure 4:**
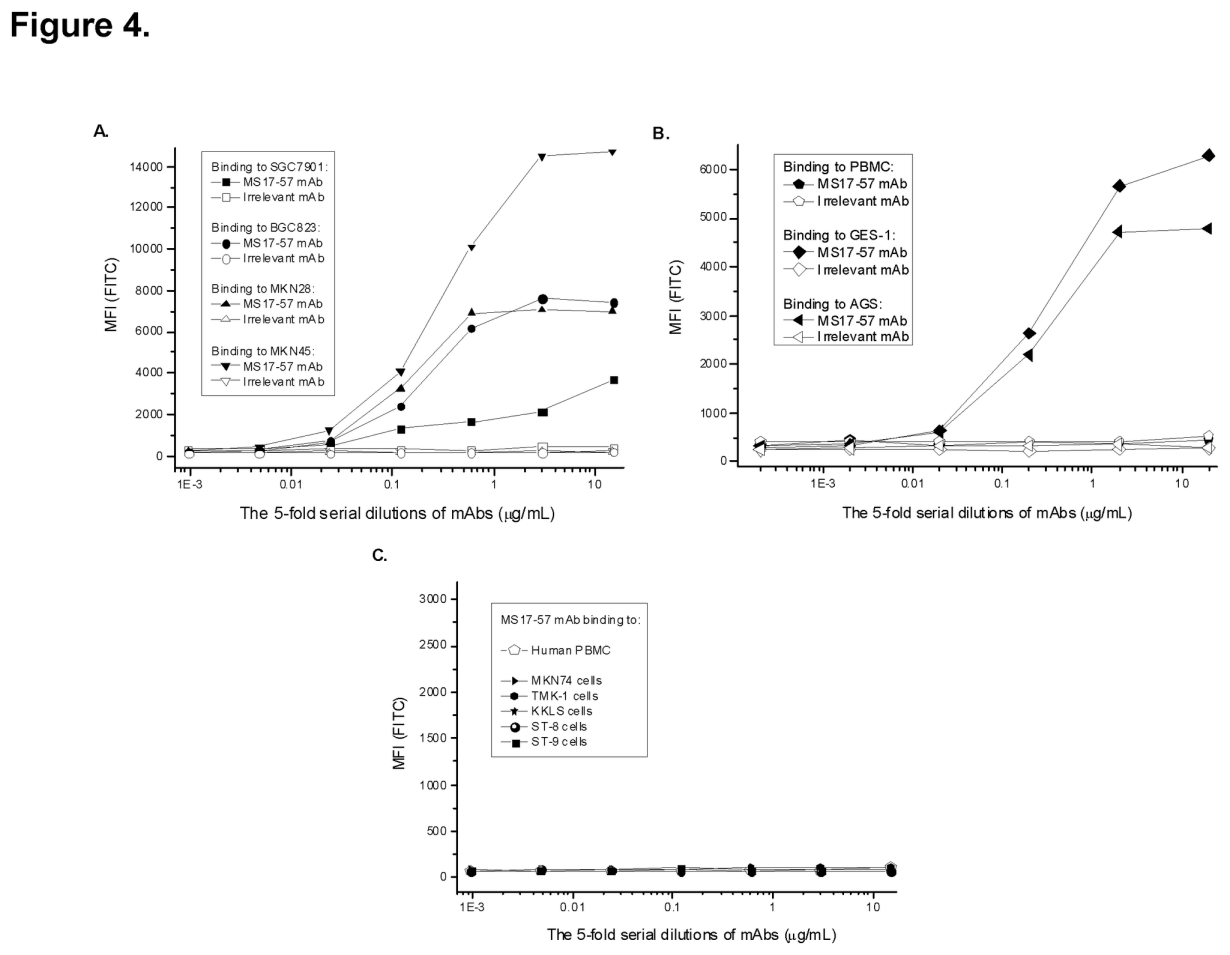
MS17-57 mAb binding to GC cells, GI cells, and human PBMCs. **A**.MS17-57 bound to all four GC cell lines that were used for live cell immunization. **B**. MS17-57 exhibited strong binding signals in GES-1 and AGS cells but no binding signal in human PBMCs. **C**. MS17-57 did not bind to human PBMCs nor any of the five GI tumor cells. Irrelevant mAb was used as the mAb isotype control.

MS17-57 bound to all four GC cell lines used for immunization, although the binding signals were not of equal intensity (Figure **4A**). The expression level of MS17-57 target was highest in MKN45 cells and lowest in SGC7901 cells. Similar to the counter-screening results, MS17-57 mAb did not bind to human PBMCs. It did bind to some other types of GC cells (Figure **4B**), but not to GI cells (Figure **4C**). Thus, the target(s) of MS17-57 are not universally expressed, although they appear to be more common in GC cells than GI cells.

**Figure 5:**
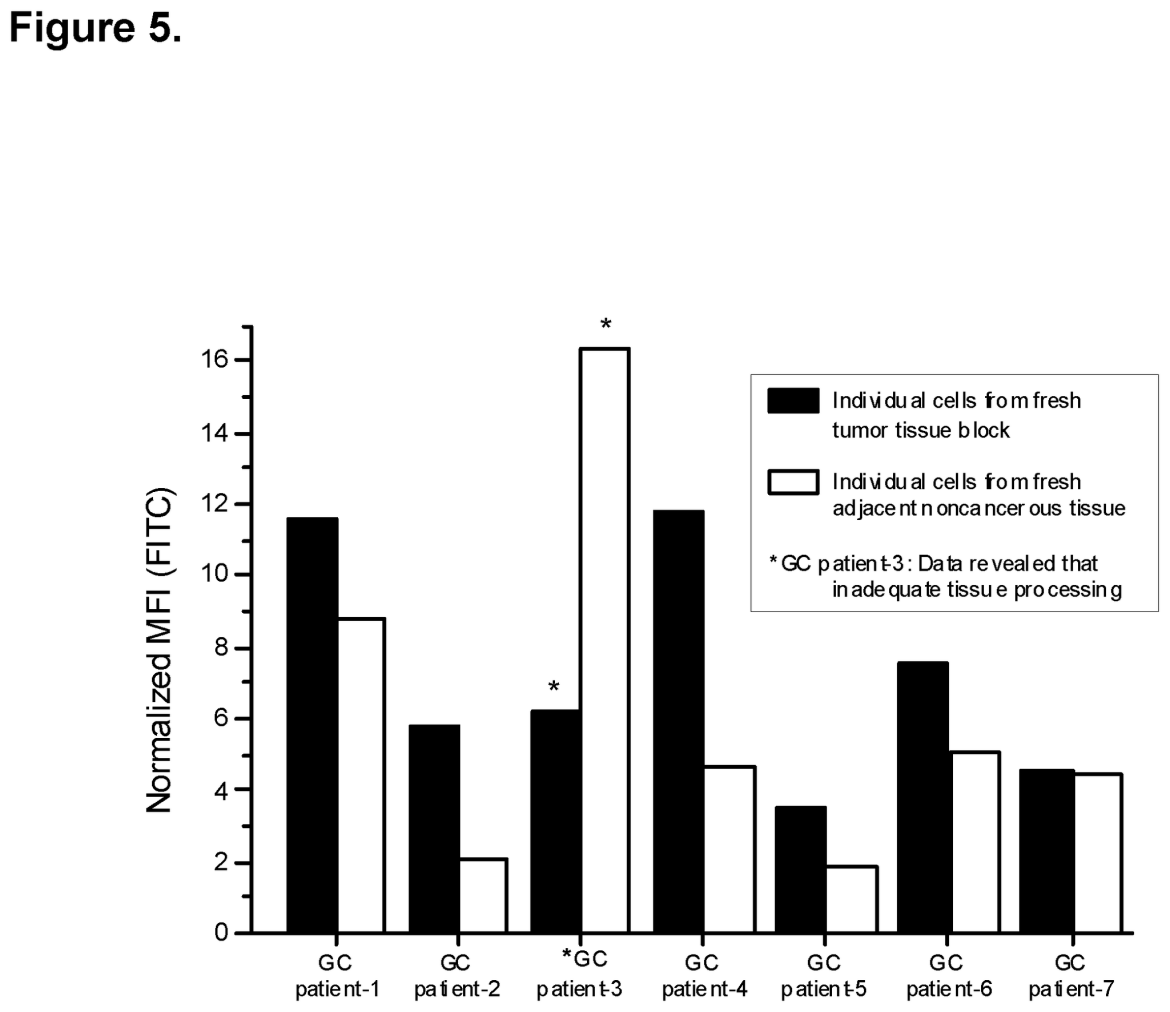
Cells from fresh surgical tissues stained with MS17-57 and isotype control mAb. Immunofluorescence cell staining with MS17-57 revealed significantly stronger staining of GI tumor tissues than that of normal (adjacent noncancerous) control tissues (*P*<0.03 overall). (Data from patient 3 were omitted for analyses because the tissue had not been properly prepared.).

Fresh tumor tissues and adjacent noncancerous tissues from six GC patients were stained for MS17-57 and isotype control mAb and then quantified with dose dependent binding in FACS-HTS. Overall, the binding signal of MS17-57 was stronger in tumor tissues than in noncancerous tissues (*P*<0.03) (Table **1** and Figure **5**). This experiment demonstrated that MS17-57 can bind to its targets in their native form on the surface of fresh tumor samples.

**Table 1:** Results of immunofluorescence cell staining, as measured with FACS, of fresh tumor and adjacent noncancerous tissues from GC patients with MS17-57 and isotype control antibody^1^.

**GC Patient**	**Tissue**	**mAb**	**MFI**	**% Stained Peak**	**Subtracted MFI^2^**	**Normalized MFI^3^**
Patient-1	Tumor	Isotype Ctrl	6.15	0.51	13.06	**11.56**
		MS17-57	19.21	18.23		
	Normal	Isotype Ctrl	11.62	0.32	15.89	**8.76**
		MS17-57	27.51	11.58		
Patient-2	Tumor	Isotype Ctrl	10.96	2.83	6.15	**5.78**
		MS17-57	17.11	11.94		
	Normal	Isotype Ctrl	28.31	10.48	-12.59	**2.06**
		MS17-57	15.72	8.26		
Patient-3*	Tumor	Isotype Ctrl	**-**	**-**	**-**	**-**
		MS17-57	**-**	**-**		
	Normal	Isotype Ctrl	**-**	**-**	**-**	**-**
		MS17-57	**-**	**-**		
Patient-4	Tumor	Isotype Ctrl	3.76	0.1	8.27	**11.83**
		MS17-57	12.03	13		
	Normal	Isotype Ctrl	4.9	0.86	1.29	**4.67**
		MS17-57	6.19	3.82		
Patient-5	Tumor	Isotype Ctrl	8.58	3.2	-0.5	**3.48**
		MS17-57	8.08	4.02		
	Normal	Isotype Ctrl	9.51	5.32	-4.65	**1.89**
		MS17-57	4.86	1.08		
Patient-6	Tumor	Isotype Ctrl	7.9	5.48	8.31	**7.59**
		MS17-57	16.21	7.63		
	Normal	Isotype Ctrl	10.33	10.21	3.84	**5.08**
		MS17-57	14.17	13.89		
Patient-7	Tumor	Isotype Ctrl	9.74	4.2	2.2	**4.54**
		MS17-57	11.94	8.16		
	Normal	Isotype Ctrl	5.45	1.81	1.17	**4.49**
		MS17-57	6.62	1.5		

^1^ Overall, normalized MFI was significantly higher in tumor tissues than in adjacent noncancerous tissues (*P*<0.03);

^2^ Calculated as (MFI_MS17-57_ − MFI_Isotype Control mAb_).

^3^ Calculated as ([parameter*X* / MFI_Isotype Control mAb_] × MFI_MS17-57_). Here parameter *X*= 3.7.

* Data from patient 3 were omitted for analyses because the tissue had not been properly prepared.)

**Figure 6:**
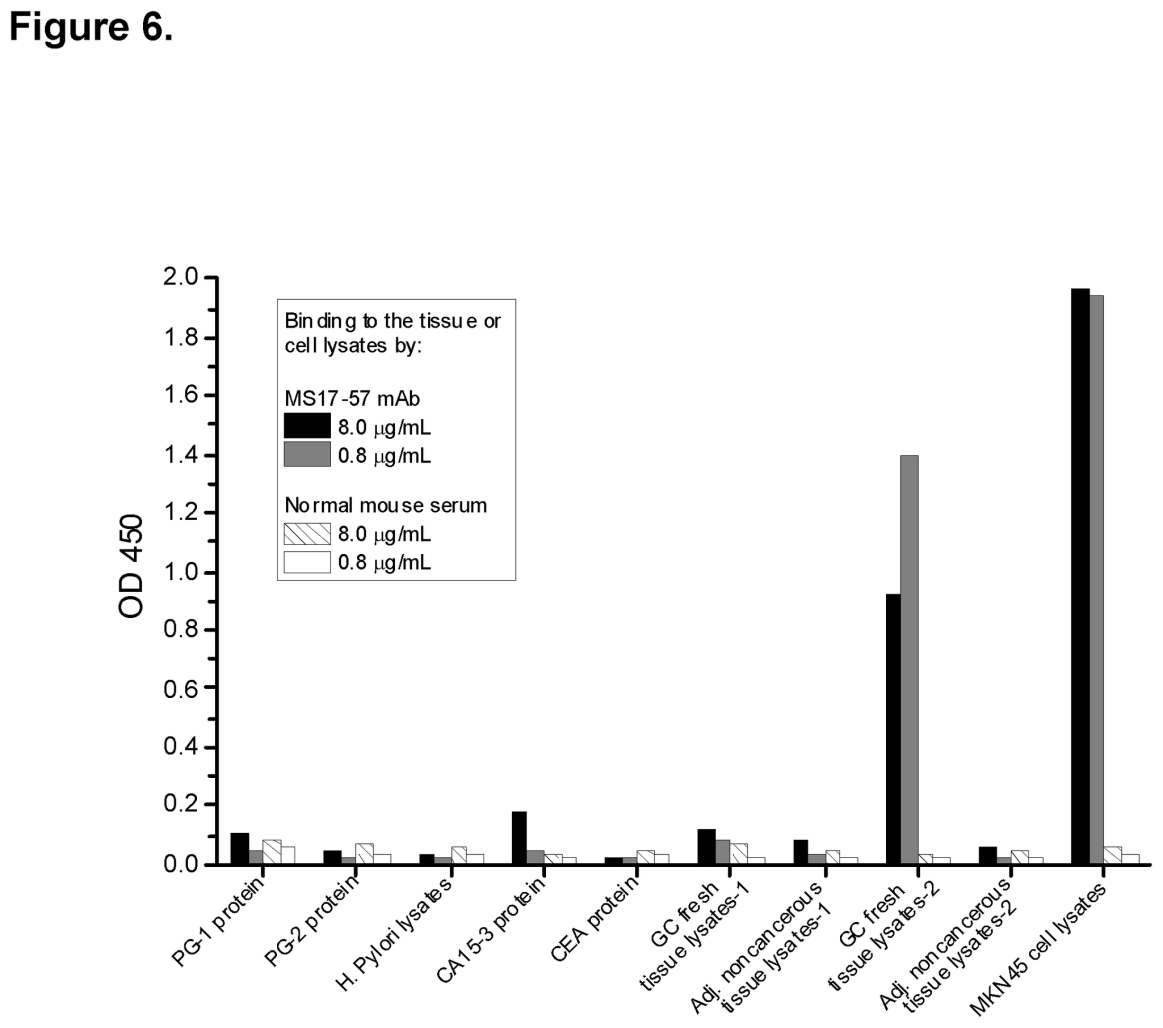
Binding of MS17-57 to purified GI cancer markers and lysates of fresh tissues and cells. ELISA results showed that MS17-57 bound to lysates of fresh GC tissues (strong binding in lysate from one patient and moderate binding in lysate from another patient) and to lysates of GC MKN45 cells, but not to fresh lysates of adjacent noncancerous tissues from the same patients. MS17-57 bound slightly to the purified CA15-3 protein but not to proteins or lysates of PG-1, PG-2, CEA, or *H. pylori*. ELISA used two dose-dependent dilutions of antibodies. Normal mouse serum protein was used as a negative control.

**Figure 7:**
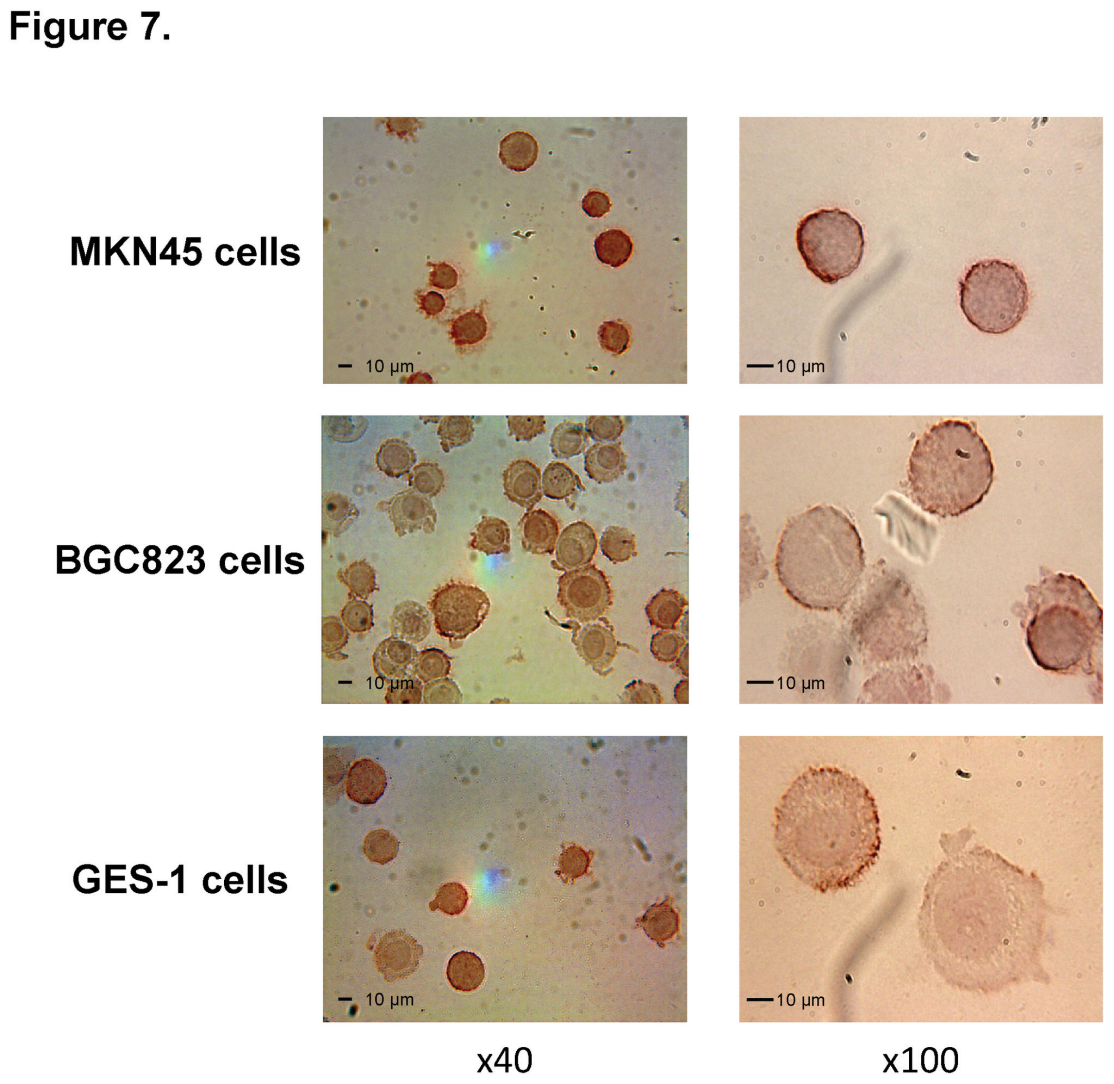
ICC staining for MS17-57 binding to MKN45, BGC823, and GES-1 cells on cytospin slides. Two ICC assays were performed; photomicrographs from one are shown at 40x and images from the other at 100x). MS17-57 bound to all three types of cells. The binding target (marker) was located on the cell surface. Images of blank and negative (isotype) controls were also obtained but are not shown here.

Another MS17-57 binding analysis was performed to purified proteins (some GI cancer markers, lysates of fresh tissues, and cells in ELISA), which demonstrated that this mAb binds specifically to target(s) on GC cells (Figure **6**). The MS17-57 target(s) might have different features than those of GI tumor-associated Ags [22,23] or other proteins (e.g., CEA [24], CA15-3 [25], PG-1 (pepsinogen-1), PG-2 [26], and *Helicobacter pylori* lysates [27]). The MS17-57 targets are at least highly expressed on the cell surface of MKN45 and BGC823 GC cells and GES-1 gastric transformed cells (Figure **7**). 

### MS17-57 Functionality against GC Cancers *in vitro* and *in vivo*

**Figure 8:**
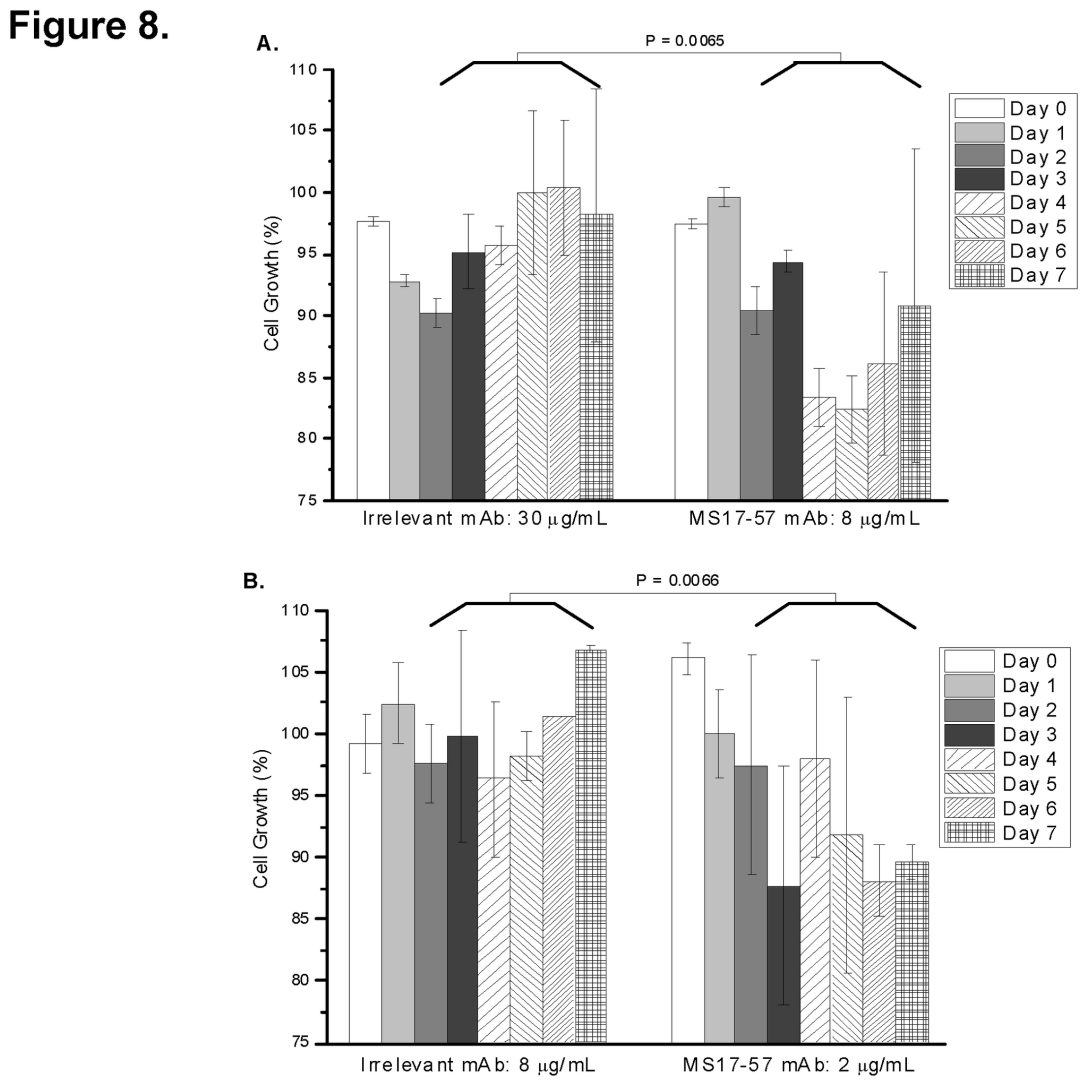
MS17-57 inhibits BGC823 and MKN45 cell growth. MS17-57 was added to BGC823 cells (8 µg/mL per well) (**A**) or MKN45 cells (2 µg/mL per well) (**B**) on 96-well tissue culture plates at day0. MS17-57 inhibited BGC823 cell growth by about 27.5% for up to 5 days and MKN45 cell growth by about 22.5% for up to 7 days. Irrelevant mAb, used for isotype control, was applied at concentrations about four times higher than that used for MS17-57. There are a significant difference of the percentage of cell growth between irrelevant mAb group (**A**) and MS17-57 group (**B**), and both statistical testing *P* values from day 3 to day 7 were 0.0065 and 0.0066.

In a variety binding assays MS17-57 specifically binds to GI cancer cell lines and does not bind to human PBMCs (normal control), which suggests that this mAb binds to GI tumor cell surface biomarkers. A cancer biomarker binder is a useful agent for detecting cancer, but it remains to be seen whether MS17-57 could be useful as a therapeutic agent. Compared with irrelevant mAb (isotype control), MS17-57 inhibited the proliferation of BGC823 cells (Figure **8A**) and MKN45 cells (Figure **8B**) (GC cell lines from the 4 cell lines used in live cell immunization), in tissue culture by approximately 32 ± 8% after 7 days of cell growth. Although the variations in some experiments were large, especially between 5 and 7 days, the assay was performed 5 times with qualitatively similar results. Thus there was an overall trend in cell inhibition by MS17-57 compared with irrelevant mAb. Inhibition of MKN45 cell growth by MS17-57 required just one dose on the first day, while late regrowth of BGC823 cell suggests that a second administration of MS17-57 at day 4 may be necessary to maintain growth inhibition for this cell line (data not shown).

**Figure 9:**
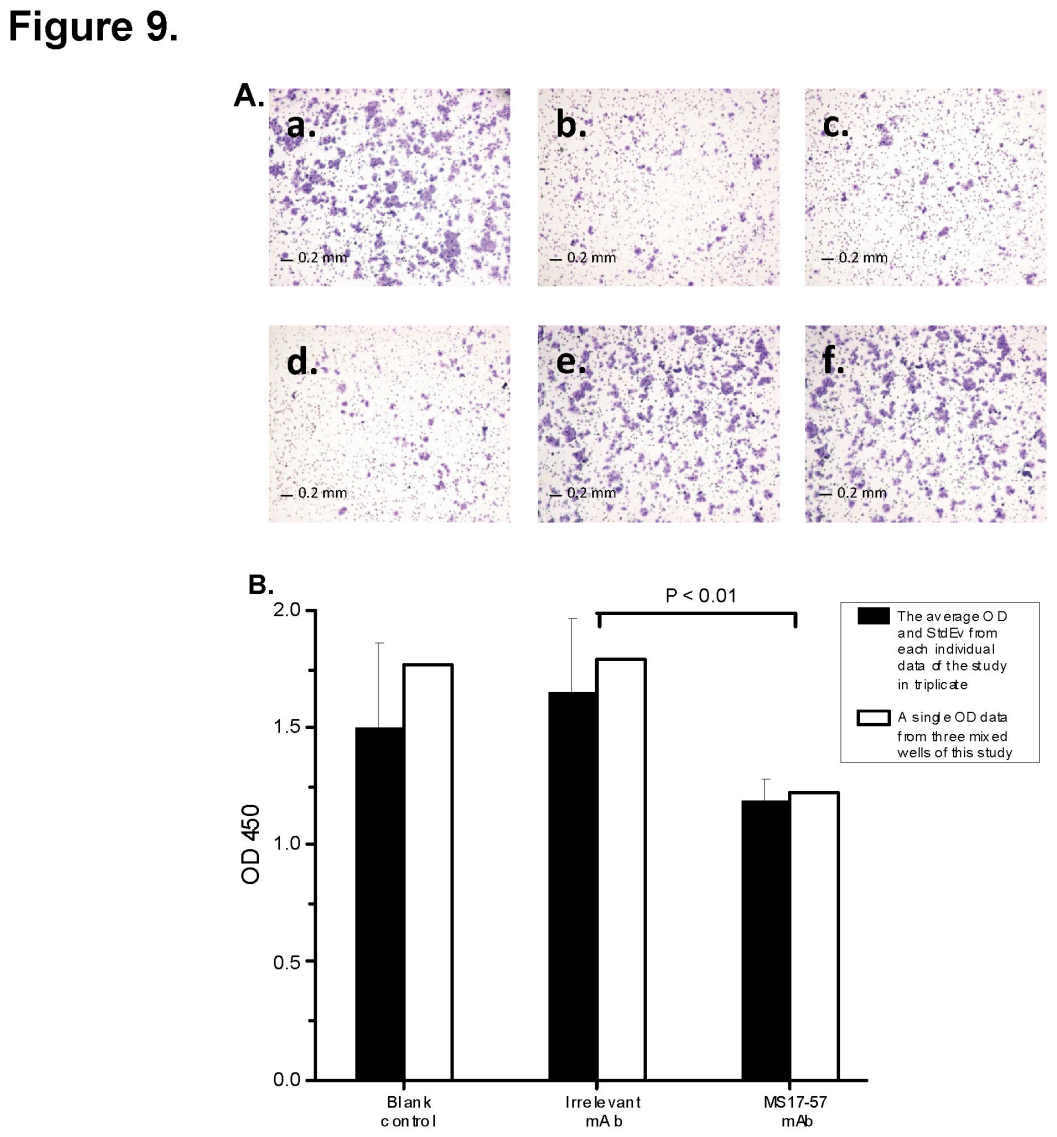
MS17-57 inhibits BGC823 cell migration over 3 days. **A**. Dose-dependent MS17-57 versus isotype mAb (irrelevant mAb) and medium controls inhibit BGC823 cell migration in a colorimetric cell migration assay. **a**: Cell culture medium control; **b**–**d**: 5, 10, and 20µg/mL MS17-57, respectively; **e and f**: 5 and 20 µg/mL irrelevant mAb, respectively. Each condition was tested in triplicate. **B**. Trypan blue dye was used to stain wells with medium control mAb, 5 µg/mL irrelevant mAb, or 5 µg/mL MS17-57 in a separated QCM colorimetric cell migration assay. Note that mean OD and standard deviation (StdEv) for each well were calculated differently. Each well was tested in triplicate. **Black bars**: mean OD and standard deviation from each individual well of the stained cells. **White bars**: single OD from three mixed wells of the stained cells. There is a significant difference of the migration ODs between irrelevant and MS17-57 mAbs and *P*<0.01.

A migration assay was conducted to determine whether MS17-57 could inhibit migration of BGC823 cells (Figure **9**) or MKN45 cells (data not shown) from moving down a transwell membrane. Comparison of the medium control and two doses of 5 and 20 µg/mL irrelevant mAb, 5, 10, or 20 µg/mL MS17-57 demonstrated migration inhibition for BGC823 cells (Figure **9A**). Another migration data were plotted from a separate experiment (Figure **9B**). Using blank control as 100% cell migration, MS17-57 inhibited cell migration by about 25 ± 5%.

**Figure 10:**
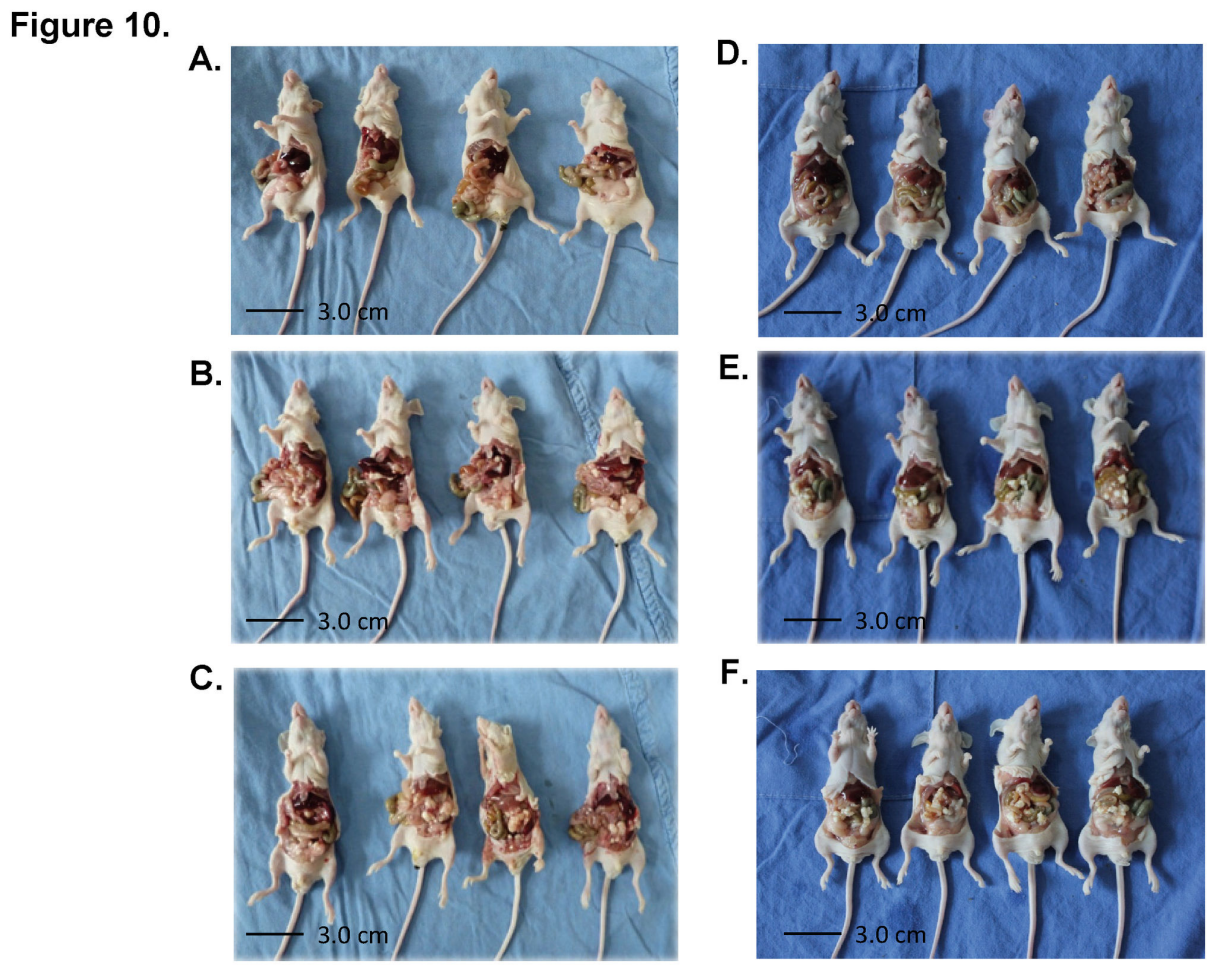
MS17-57 inhibits tumor growth in nude mice. (**A**) MS17-57 mAb inhibits the growth of MKN45 tumor cell xenografts. Cells (1x10^6^) mixed with 100 µg of MS17-57 mAb (50 µg/mL in PBS) were injected i.p. into the abdominal cavity of male nude mice and 4 mice in each group. About 6 weeks later, mice with a palpable tumor were counted for an average of 1.5 node/each nude mouse and an average tumor diameter 0.3 cm; (**B**) The same procedures as in (A) using MKN45 cells in nude mice but with irrelevant mAb and a final count of an average of 8.5 node/each nude mouse and an average tumor diameter of about 0.31 cm; (**C**) The same procedures as (A) using MKN45 cells in nude mice but with PBS (buffer) and the final tumor nodes count an average of 9.0 node/each nude mouse and an average tumor diameter of about 0.28 cm; (**D**) The same procedures as (A) but using BGC823 cells in nude mice with MS17-57 mAb and a final tumor nodes count of an average of 1.0 node/each nude mouse and an average tumor diameter of about 0.27 cm; (**E**) The same procedures as (B) but using BGC823 cells in nude mice with irrelevant mAb and a final tumor nodes count of an average of 7.0 node/each nude mouse and an average tumor diameter of about 0.31 cm; (**F**) The same procedures as (C) but using BGC823 cells in nude mice with PBS and a final tumor nodes count of an average of 6.5 node/each nude mouse and an average tumor diameter of about 0.30 cm.

In a preliminary in vivo study, MS17-57 inhibited tumor growth from MKN45 cells and BGC823 cells that were selected from 4 GC cell lines in inoculated JAX nude mice (Figure **10**). The experimental design was the same as that of the in vitro studies using medium control and irrelevant mAb controls. The average size of tumor nodules was not significantly difference between the control groups and MS17-57 group, but the average number of tumor nodules were 7.75 versus 1.25 (irrelevant mAb versus MS17-57) that have the significant differences.

### PALP and IALP are MS17-57 Targets

**Figure 11:**
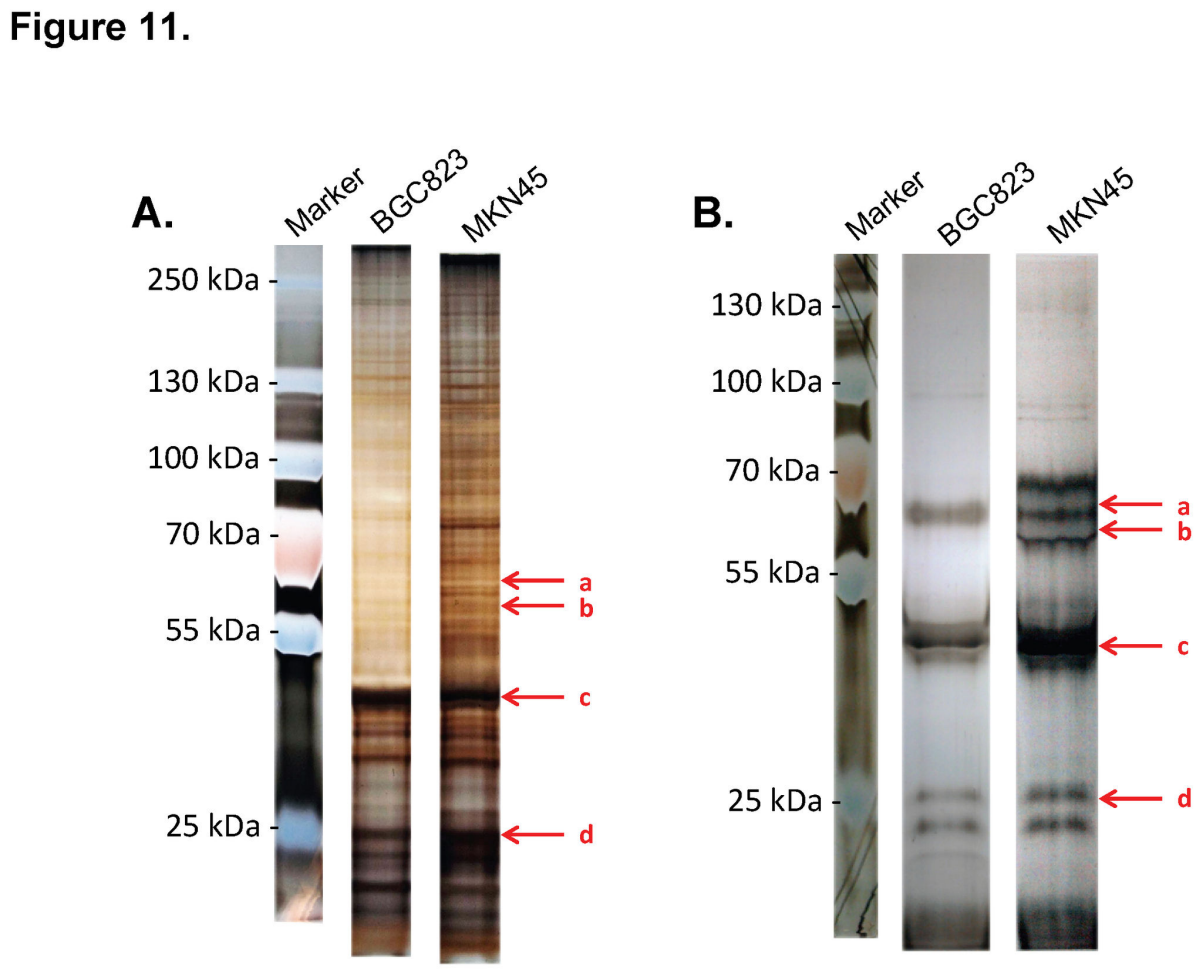
Pull-down assay for MS17-57 targets from cellular lysates of BGC823 and MKN45 cell lines. MS17-57 mAb can pull down the targets from the cellular lysates of both BGC823 and MKN45 cell lines in direct IP (**A**) and indirect IP (**B**) assays. **a**: PALP (MW = 57.4 kDa); **b**: IALP (MW = 56.8 kDa); **c**: heavy chain of denatured MS17-57 (MW = 50 kDa); **d**: light chain of denatured MS17-57. Note the scale is different for the direct and indirect IP gels.

Western blotting was used to determine the molecular weights of the MS17-57 targets (data not shown), which demonstrated one band from BGC823 lysates (about 58 kDa) and two bands from MKN45 lysates (about 58 kDa and 56.5 kDa). Pull-down assays with indirect IP (Figure **11A**) and direct IP (Figure **11B**) were conducted to define the targets of MS17-57. Many combinations of labeling, conjugation and eluting conditions, buffer systems, cell lysates, and processing procedures were used (description of these conditions not shown). Direct IP revealed two target bands of about 58 kDa and 56.5 kDa on MKN45 cell lysates and one band of about 58 kDa on BGC823 cell lysates (Figure **11B**). Other bands on these images were the heavy and light chains of denatured MS17-57.The bands on direct IP SDS-PAGE gels (Figure **11B**) were cut and sent for mass spectrometry (MS) analysis. The MS analytic results of MS17-57 binding targets revealed scores of above 95% (determined by MS software) for both PALP and IALP in MKN45 lysates and for PALP in BGC823 lysates (data not shown). ELISA experiments confirmed that MS17-57 was binding to purified PALP and IALP proteins (R&D Systems, Minneapolis, MN, USA) (data not shown).

**Figure 12:**
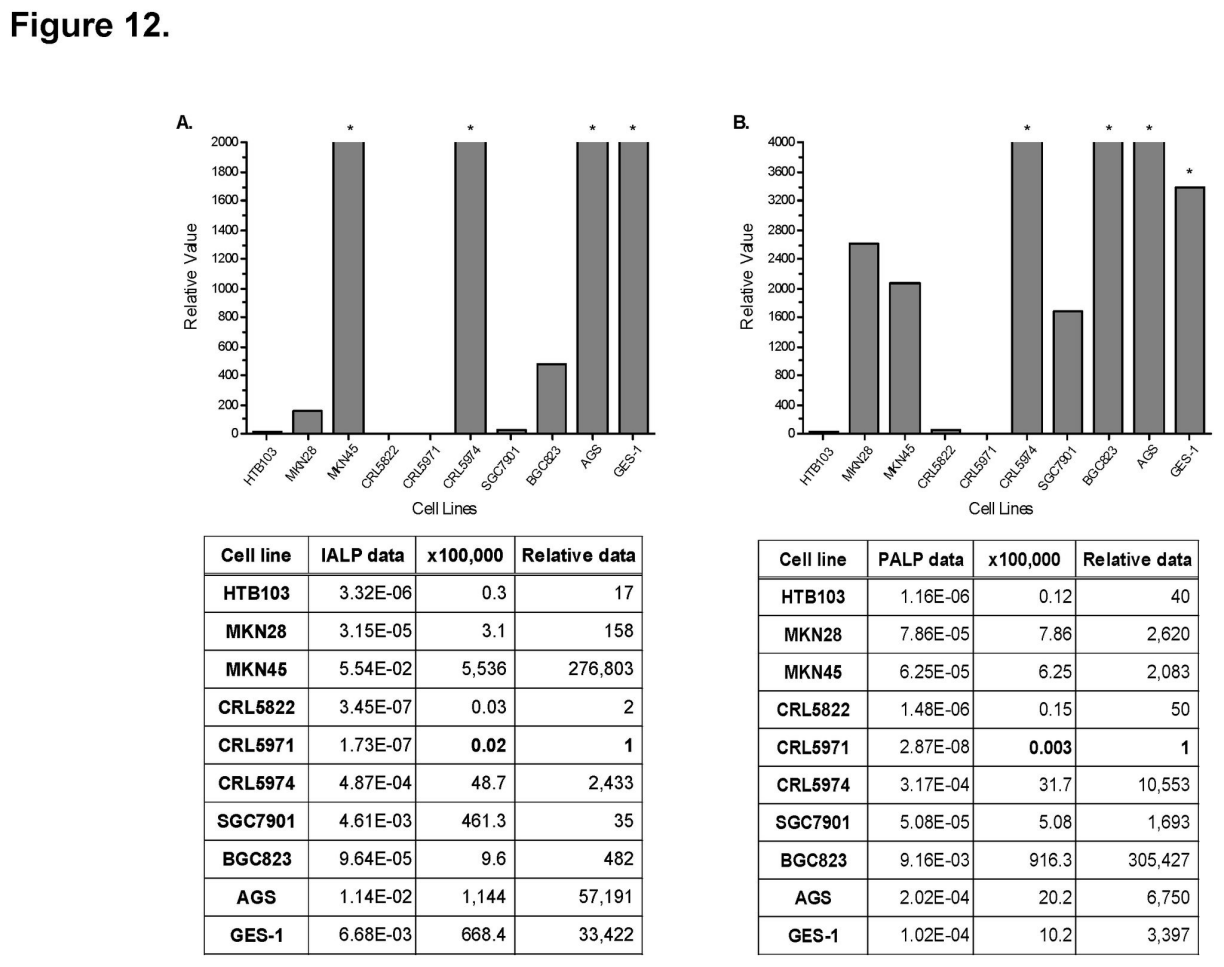
IALP and PALP mRNA expression levels in 10 cell lines by qRT-PCR analysis. mRNA expression of IALP (**A**) and PALP (**B**) was the highest in MKN45, CRL5974, AGS, and GES-1 cell lines. In BGC823 cells, mRNA expression was much higher for PALP than IALP.*Superelevated values compared with average values with qRT-PCR.

We used qRT-PCR to analyze the mRNA expression levels of PALP and IALP from lysates of 10 GI tumor cell lines. Expression levels varied greatly. IALP was most strongly expressed in MKN45 cell lysate (Figure **12A**), and PALP in BGC823 and MKN45 cell lysates (Figure **12B**). The expression level of PALP was more than 100 times higher in BGC823 cells than MKN45 cells. These data are in line with results from our IP and MS analysis.

## Discussion

We developed and described the mAb MS17-57 and identified PALP and IALP as its targets. These targets were ectopically expressed on the surface of GC cells. The functions of MS17-57 on GI cancer were confirmed both in vitro and in vivo studies [8]. 

A potential therapeutic lead for GI cancer therapy, MS17-57 was generated by immunization with live GC cells and the unique use of FACS-HTS. The immune responses in sera of immunized mice were determined using titration ELISA. The binding signals of GC cells and human PBMCs from sera were of similar strength, but one of these sera was absorbed [28] by the lysates of human PBMC, then the binding signals in ELISA were much higher to the lysates of GC cells than it to PBMCs (data not shown). We are not sure whether MS17-57 binds to the targets (PALP and IALP) as a linear epitope or a conformational epitope, since the mAb could bind to the protein in both lysates and live cells. The mAb was captured by live cell screening with FACS-HTS rather than by screening with ELISA-HTS or other assays [29,30] using proteins in cell or tissue lysates. One planned future investigation will involve binding of MS17-57 to ALPs, including PALP and IALP, in protein modeling and crystal structure experiments; the results should reveal the binding motif and features of protein-protein interactions.

ALP is a hydrolase that cleavages phosphate groups from primary or secondary metabolites in vivo [31,32], including nucleotides, proteins, and alkaloids. Researchers have studied the role of ALP in inflammation and metabolic disease [33-35]. Hypophosphatasia features selective deficiency of activity of the tissue-nonspecific (liver, bone, or kidney) ALP isoenzyme; PALP and IALP isoenzyme activities are not reduced [36]. Hypophosphatasia is a hereditary disease characterized by low activity of total serum ALP accompanied by a range of skeletal diseases. The main circulating ALP isoenzymes (bone ALP, liver ALP, IALP, and PALP) are in six families with hypophosphatasia [37]. Six ALP families are known, including PALP and IALP (a version of PALP that lacks the last 24 amino acids at the C terminal and is encoded by the *IALP* gene). Most ALP isoenzymes, including PALP and IALP, are secreted from many types of cells. Yohsinaru et al. reported that the expression of glycosylphosphatidylinositol-anchored carcinoembryonic Ag (CEA) and ALP on the cell surface of a variety of cancer cell lines and a lung diploid cell line (WI38) upon exposure of the cell lines to a cell differentiation agent (sodium butyrate) to induce cell differentiation and expression of the two tumor-associated Ags [38]. The mechanism and function of ALP expressed on the cell surface are not clear, although it is ectopically expressed in cancer cell lines.

The fact that MS17-57 inhibited the growth, proliferation, and migration of GC cells suggests that this mAb could be the basis for a therapeutic agent for cancer treatment and prevention of metastasis. MS17-57 bound to not only GI tumor tissues and cells but also to transformed GI cells (i.e., GES-1), which means the mAb could bind to the target(s) expressed at a relatively early stage of cancer development. The levels of PALP and IALP ectopically expressed on the cell surface were not balanced among these GI cancer cells. Because the level of ALPs expressed may or may not affect the development of cancer, to help define the targets of MS17-57, we will conduct functional proteomics reverse-phase protein assay for ALPs in the intracellular signaling pathways.

In a preliminary study, MS17-57 mAb inhibited tumor growth in a mouse model. We will follow up these promising results by investigating the *in vivo* function of MS17-57 using additional approaches including other strains of mice, the tumor inoculation methods, and alternative metastatic models. 

In summary, we generated the mAb MS17-57 by using the unique FACS-HTS and identified its targets, PALP and IALP, which were ectopically expressed in the extracellular matrix of GI cancers. This mAb inhibited GC cell proliferation and prevented their migration in preliminary *in vitro* and *in vivo* studies. MS17-57 could be an example of cancer biomarkers identification leading to promising therapeutic targets through mAb generation using our unique HTS technology.
